# Applications of artificial intelligence in non–small cell lung cancer: from precision diagnosis to personalized prognosis and therapy

**DOI:** 10.1186/s12967-025-07591-z

**Published:** 2025-12-23

**Authors:** Luyuan Chang, Haipeng Li, Wenzong Wu, Xinyu Liu, Jiaqi Yan, Zuo Chen, Huan Wu, Shilong Song

**Affiliations:** 1 The First Department of Clinical Medicine (First Affiliated Hospital), Bengbu Medical University, Bengbu, Anhui China; 2 Department Mental Health, Bengbu Medical University, Bengbu, Anhui China; 3 The Department of Radiotherapy of the First Affiliated Hospital of Bengbu Medical University, Bengbu, Anhui China

**Keywords:** Non–small cell lung cancer (NSCLC), Artificial intelligence (AI), Precision diagnosis, Personalized prognosis, Treatment decision support

## Abstract

**Background:**

Non-small cell lung cancer (NSCLC) carries a major global burden. The rapid growth of multimodal medical data challenges conventional methods to deliver stable, transferable and interpretable decisions across heterogeneous longitudinal high dimensional inputs.

**Methods:**

This review summarizes advances in artificial intelligence (AI) for NSCLC from 2023 to 2025 and outlines a translation-focused framework that links algorithmic progress to clinical utility. We survey thoracic imaging, digital pathology and multiomics together with evaluation practices and implementation guidance. We also adopt a critical perspective.

**Results:**

Many high performing deep models remain black boxes, and popular post hoc explanations such as Grad CAM heatmaps are rarely validated for faithfulness or stability, which undermines clinician trust and limits use in high stakes decisions. To address this gap, we propose a minimum evidence package for explainability that comprises sanity checks, quantitative faithfulness tests such as deletion or insertion, ROAR or IROF and infidelity, stability analyses, concept level validation for example TCAV with statistical testing, and prospective human factors studies that demonstrate improved decisions without automation bias. Across modalities, evaluation has expanded beyond discrimination to include calibration, uncertainty quantification (UQ) and subgroup analyses across scanners, sites and populations. However, the evidence base remains constrained by retrospective single center designs, inconsistent external or temporal validation and limited decision curve analysis (DCA). Translational priorities include a staged validation ladder from technical to clinical to prospective deployment, alignment with Software as a Medical Device frameworks, interoperable governance, fairness and economic assessment, and validated explainability coupled with uncertainty aware selective workflows.

**Conclusions:**

Looking ahead, progress will depend on multimodal foundation models, causal and temporal modeling, and regulatory qualification of computable biomarkers with verified explanations, supported by multicenter prospective studies that demonstrate durable generalizability, clinical value and clinician trust.

## Introduction

Lung cancer remains the leading cause of cancer death worldwide. According to GLOBOCAN 2022, about 2.48 million new cases and 1.82 million deaths occurred globally, making lung cancer both highly incident and the leading cause of cancer mortality in many regions [1]. Non-small cell lung cancer (NSCLC) accounts for 80–85% of cases and comprises adenocarcinoma, squamous cell carcinoma, and large-cell carcinoma [2]. Despite advances in low-dose computed tomography (LDCT) screening, perioperative immuno-oncology, targeted therapies, and stereotactic radiotherapy, population outcomes remain suboptimal [2–4]. Recent SEER/ACS estimates show an overall 5-year relative survival of about 32% for NSCLC, with pronounced stage gradients: localized about 67%, regional about 40%, and distant about 12% [5]. Poor outcomes reflect late presentation, substantial inter- and intratumoral heterogeneity, and primary or acquired resistance to systemic therapy [6, 7]. These challenges strain traditional diagnostic and prognostic approaches, which struggle to integrate high-dimensional, multimodal information, including imaging, whole-slide histopathology, genomic and transcriptomic profiles, longitudinal clinical data, and environmental exposures, into actionable decisions [8, 9].

Heterogeneity and evolutionary dynamics are central to the biology of NSCLC [6, 7]. Spatially distinct subclones, variable target expression, divergent microenvironmental niches—including inflamed, excluded, and desert phenotypes—and shifting therapeutic selective pressures produce complex response patterns and resistance [10–13]. Environmental exposures, most notably fine particulate matter PM2.5, contribute to lung adenocarcinoma, particularly among never-smokers, and interact with molecular drivers such as the epidermal growth factor receptor (EGFR) [14]. This clinical, biological, and environmental heterogeneity motivates methods that learn structure across scales and generate individualized predictions that update over time [8, 15].

Artificial intelligence (AI), particularly machine learning (ML) and deep learning (DL), has progressed from proof of concept to multicenter and early prospective evaluations across oncology workflows [16, 17]. In thoracic imaging, DL systems trained on LDCT and diagnostic CT achieve expert level performance in nodule detection and malignancy risk estimation. They are under prospective evaluation for longitudinal risk prediction in screening cohorts, for example models that predict one to six years of lung cancer risk from a single LDCT [18, 19]. In digital pathology, self supervised foundation models and multiple instance learning (MIL) standardize histologic subtyping, quantify immunohistochemistry (IHC) such as the programmed death ligand 1 (PD-L1) tumor proportion score (TPS) with higher reproducibility than manual scoring, and increasingly infer actionable biomarkers such as EGFR directly from routine hematoxylin and eosin (H&E) slides [12, 20–22]. Parallel advances in multimodal fusion and representation learning enable integration of radiology, pathology, and omics with structured clinical data to support individualized prognosis and treatment selection [8, 23, 24]. Building on these modality-specific gains, we present a unifying framework for multimodal fusion [25]. We define multimodal fusion as the principled integration of imaging, digital pathology, genomics, and clinical data to improve diagnosis, prognosis, and treatment in NSCLC [26]. This review explains how fusion is realized across radiology, pathology, and omics, describes the alignment and aggregation of heterogeneous representations into coherent patient-level embeddings, and shows how fused models are integrated into routine clinical workflows to support decision-making [27]. Throughout, we highlight fusion’s contribution to generalization, interpretability, and clinical utility, and we evaluate each application with a consistent toolkit of external validation, calibration, and decision-curve analysis to enable fair, decision-relevant comparisons [28]. At the architectural level, contemporary systems include four major families. First, Transformer backbones use self attention for spatial and contextual integration [29]. Second, temporal and frequency attention, exemplified by Fourier attention (FA) and wavelet attention (WA), captures long range periodicity and multiscale transients in longitudinal imaging and circulating tumor DNA (ctDNA) time series [30–32]. Third, graph neural networks (GNNs) encode pathway and topological constraints for multi omics integration. Fourth, generative adversarial networks support denoising, super resolution, and stain or style normalization. Together with early prospective evaluations, these trends position AI as a scalable, data-driven layer that augments radiology, pathology, genomics, and clinical decision-making throughout the NSCLC care continuum [16, 33].

**Fig. 1:**
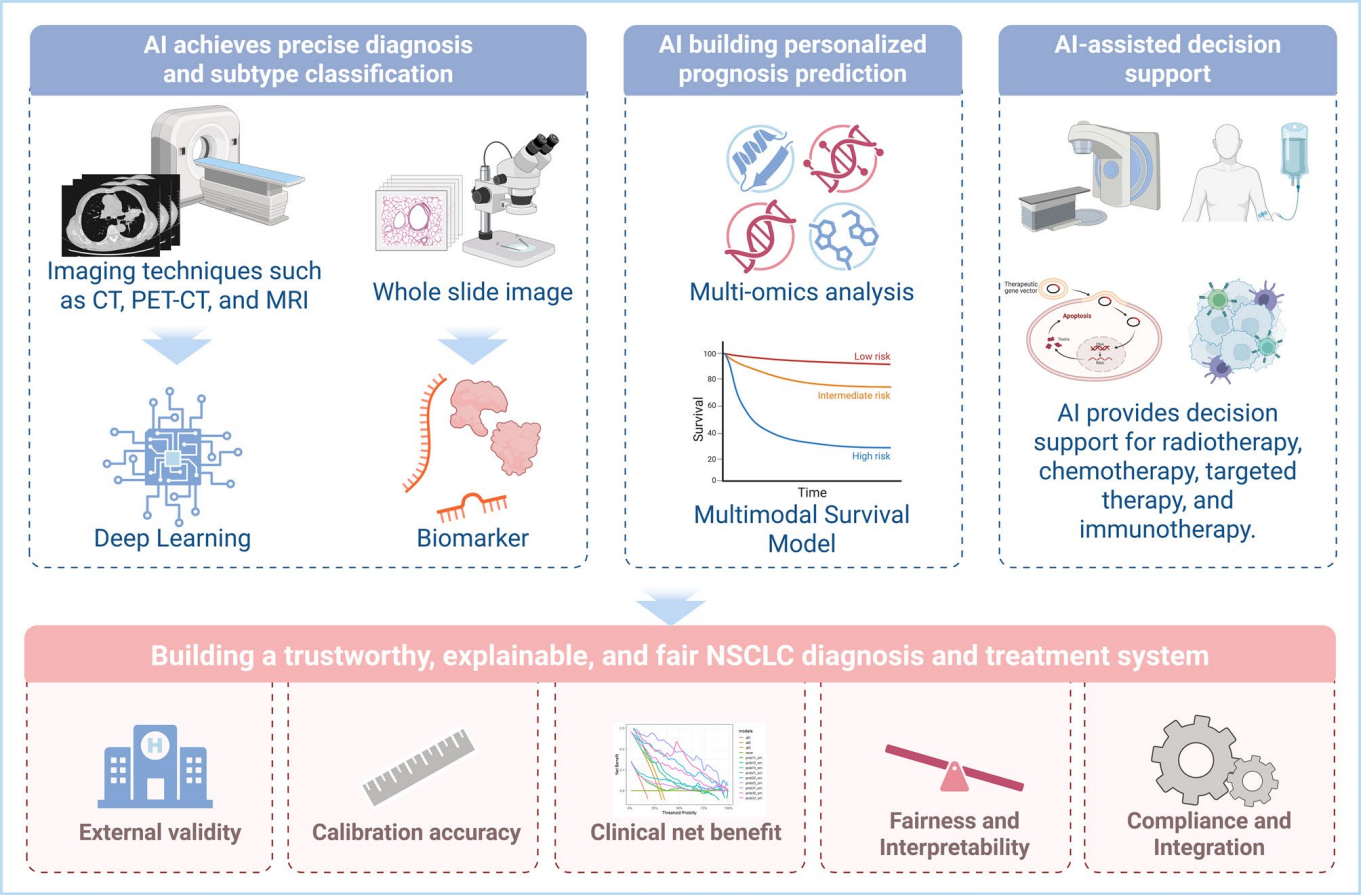
AI-enabled NSCLC pathway for precise diagnosis, personalized prognosis and clinical decision support

A targeted search of PubMed, MEDLINE, Embase, Web of Science, Scopus, and Cochrane CENTRAL identified studies published between 1 January 2023 and 17 August 2025. Search queries combined controlled vocabulary and free-text terms for NSCLC and AI across imaging, digital pathology, multi-omics, prognosis, treatment decision support, and drug discovery. Records were independently screened by two reviewers for relevance to the review objectives, with priority given to human NSCLC studies featuring clinically meaningful endpoints and implementation insights. As a narrative review, no preregistered protocol, formal risk-of-bias assessment, or quantitative synthesis was employed. When necessary to contextualize developments from 2023 to 2025, seminal prior work and select pre-2023 studies were cited. Using this methodology, we review 2023 to 2025 advances across the NSCLC pathway (Fig. 1): precision diagnosis and subtyping using CT, Positron Emission Tomography-Computed Tomography (PET-CT), and Magnetic Resonance Imaging (MRI) radiomics and deep learning [18], as well as whole-slide image biomarker inference [22]; individualized prognosis with multi-omics and multimodal survival models [34]; and decision support for radiotherapy, chemotherapy, targeted therapy, and immunotherapy, together with AI-enabled drug discovery [35]. We also highlight translational requirements, including external validity, calibration, clinical net benefit, fairness, and explainability, and we outline regulatory and workflow-integration considerations [36–38]. Finally, we propose an agenda for trustworthy, interpretable, and equitable deployment [39], including a pragmatic validation ladder and postmarket change-control and recalibration plans.

## Applications of AI in diagnosis and subtyping of NSCLC

### AIassisted imaging diagnosis (CT, PETCT, MRI; radiomics and deep learning)

In thoracic radiology, AI aims to improve sensitivity, specificity, and workflow efficiency in both screening and diagnostic evaluation [34]. Deep learning CNNs trained on chest CT detect and characterize pulmonary nodules, prioritize worklists, and generate quantitative malignancy-risk estimates, which reduces interobserver variability and mitigates reader fatigue in high-volume settings [34–36]. Nodule detection and longitudinal risk stratification are related but distinct tasks [18, 35]. End-to-end 3D CNNs for LDCT screening, exemplified by the 2019 Google system in Nature Medicine, achieved an area under the receiver operating characteristic curve (AUROC) of approximately 0.94 for per-case cancer detection and reduced false positives by about 11% and false negatives by about 5% versus expert readers in retrospective testing [38]. In parallel, the Sybil model, developed and validated across multiple centers and reported in the Journal of Clinical Oncology in 2023, predicts individual one- to six-year lung-cancer risk from a single LDCT without additional clinical covariates. External AUROCs of approximately 0.75–0.81 have been reported, and such models can inform interval scheduling and escalation pathways for high-risk individuals [18].

In addition to detection and risk stratification, DL enhances the segmentation of tumors and organs-at-risk, facilitating volumetry, growth-kinetics modeling, and radiotherapy planning [37, 39]. In PET-CT, AI aids in mediastinal staging, such as classifying nodal involvement with AUROCs around 0.90, and detecting occult metastases. In MRI, which is less commonly used for thoracic cancers, AI supports brain-metastasis surveillance and target-volume delineation [40–43]. Denoising and super-resolution techniques based on learning further enhance low-dose image quality, preserving quantitative imaging features [44, 45].

Methodologically, comparisons between radiomics and deep learning recur in thoracic oncology [46]. Handcrafted radiomics extracts shape, intensity, and texture features to infer phenotypes and biomarker surrogates such as histology, EGFR status, and invasiveness, yet it is sensitive to heterogeneity in slice thickness, reconstruction kernel, vendor, and to feature engineering choices [46, 47]. Accordingly, pipelines aligned with the Image Biomarker Standardisation Initiative (IBSI) should explicitly document voxel resampling with isotropic 1.0 mm, intensity discretization using a fixed bin size or number, pre filters such as Laplacian of Gaussian (LoG) radii, and the exact feature set; releasing code and parameter files enables independent reproducibility. By contrast, deep learning leverages voxel level signals and peritumoral context and often outperforms classical models when trained on diverse, harmonized datasets; however, both paradigms require external validation, calibration, and decision curve analysis (DCA) to demonstrate clinical utility [28, 48, 49] (Table 1). To carry these methods into practice, external validation and calibration must follow disciplined procedures. External validation and calibration: practical guidance. Lock the full analysis pipeline before any testing begins [52]. Select cohorts that are both geographically and temporally external and that span multiple vendors and protocols. Fit all preprocessing on development data only and apply it unchanged to the external cohort. Split at the patient level and keep sites and scanners separated to prevent leakage from acquisition signatures [53]. Report discrimination, calibration, and clinical utility together, and provide subgroup results by site, scanner, sex, age, ancestry, stage, and smoking status. See section“Validation, regulation, and workflow integration” for detailed checklists and sample size targets.

**Table 1:** Methodological comparison: radiomics vs deep learning

Topic	Radiomics (hand-crafted)	Deep Learning (end-to-end)	Key references
Features and inputs	Shape/intensity/texture features; sensitive to acquisition heterogeneity (slice thickness, reconstruction kernel, vendor)	Voxel-level signals plus peri-tumoral context; automatically learned multi-level representations	[46, 47]
Reproducibility and standardization	IBSI-aligned parameters: voxel resampling (e.g., 1.0 mm isotropic), intensity discretization, LoG radii; release code and parameter files	Document preprocessing/augmentation/architectures and weights; report differences between training and inference domains	[28, 47, 50]
Performance and generalization	Effective on homogeneous data but vulnerable to domain shift	Often outperforms traditional models on multi-domain/harmonized datasets	[46, 48]
Evidence and reporting	External validation, calibration curves, DCA, and stratified error analyses	Same requirements; additionally adhere to TRIPOD+AI for transparent reporting	[28, 49, 51]

Domain shift remains a central challenge for deployment [54]. Performance on external cohorts often falls relative to internal testing because of protocol and population differences, and decreases of about 5 to 10 AUROC points are common across centers [55, 56]. Mitigation strategies include, first, multi domain training across vendors and protocols; second, strong data augmentation combined with physics informed harmonization such as ComBat and kernel aware resampling [47, 50, 57]; third, out of distribution (OOD) detection with case level uncertainty estimation to flag low reliability outputs [58, 59]; and fourth, test time adaptation or augmentation to stabilize predictions at deployment. At a minimum, reports should include internal and external validation splits, calibration curves, and error analyses stratified by scanner vendor, slice thickness, reconstruction kernel, geography, sex, age, and smoking status [60]. Rigorous external validation should use a locked pipeline [61], apply preprocessing learned on development data without modification, and adopt patient splits that are separated by site and scanner, while reporting discrimination, calibration, and decision analysis together; section“Validation, regulation, and workflow integration” provides an execution protocol that reduces optimistic bias and improves transportability [62].

Several nodule management solutions have received regulatory clearance, bridging research evidence and deployable clinical tools [63, 64]. Examples include Riverain ClearRead CT with FDA 510k clearance in the United States and Veye Lung Nodules with an EU MDR CE mark, which function as concurrent readers for detection, volumetry, and growth assessment in PACS integrated workflows [65–67]. Real world rollouts emphasize additional requirements: seamless RIS and PACS integration, stable inference latency, auditable trails, and collaboration between humans and AI such as AI triaged worklists and structured reports that state uncertainty explicitly [68, 69]. Finally, for each imaging AI task including detection, risk stratification, staging, invasiveness, and risk of metastasis, studies should prespecify outcomes and thresholds, report calibration including flexible calibration plots and DCA also called DCA, provide subgroup analyses by vendor, protocol, and key demographic and clinical factors, and release IBSI conformant parameters together with reference implementations to enable independent verification [70].

Heatmaps and saliency maps used to explain nodule detection or malignancy risk do not provide evidence of model reasoning unless validated [71]. These maps can remain stable when labels or weights are randomized, highlight scanner-specific artifacts such as kernel edges, beam-hardening, or bed/table contours, or change significantly with minor preprocessing adjustments [72]. For each imaging task, we recommend reporting the following: sanity checks where saliency should collapse with label or weight randomization, faithfulness metrics such as deletion or insertion curves or ROAR/IROF showing monotonic performance loss when removing important regions compared to matched controls, stability across seeds, augmentations, and scanners, and clinical alignment through concept-level tests such as spiculation or necrosis masks with effect sizes and confidence intervals [73]. Explanations should be accompanied by uncertainty and selective deferral, especially near decision thresholds used for escalation pathways. They must also be evaluated for automation bias in reader studies [74].

### AI in pathological subtyping and biomarker inference

Digital pathology is another frontier where AI is transforming the diagnosis of NSCLC [15, 20]. Traditionally, histologic subtyping and the identification of molecular alterations require meticulous microscopy and multiple ancillary assays, such as IHC for TTF-1 and p40 and fluorescence in situ hybridization or sequencing for genomic alterations [75]. AI analyzes whole slide images (WSI) of tumor tissue to automate and augment these tasks. Deep learning models trained on labeled H&E slides accurately classify NSCLC histopathology, distinguishing adenocarcinoma from squamous carcinoma and other subtypes, with performance comparable to expert pathologists. For example, a 2020 study reported accuracy of approximately 0.95 for differentiating adenocarcinoma from squamous carcinoma on WSIs [76]. WSI pipelines use MIL with attention pooling. Tile level embeddings are aggregated via self attention or gated attention to produce slide level predictions. Recent variants apply token based Transformers to tile sequences with two dimensional positional encodings. We record the reported layer count, number of attention heads, and whether frequency aware attention such as FA or WA, or stain aware modules, was used to improve robustness. For IHC quantification, lightweight Vision Transformer heads operating on nucleus or patch tokens are increasingly used to standardize the PD-L1 TPS [29]. A particularly promising application is molecular virtual staining, namely biomarker prediction directly from routine H&E histology [20, 77]. Building on foundational work such as that by Coudray and colleagues, who reported an AUROC of approximately 0.82 for EGFR prediction from H&E, Campanella and colleagues in 2025 developed EAGLE, a refined pathology AI that was prospectively evaluated in a real world setting for EGFR prescreening [22]. In Nature Medicine, EAGLE was trained on more than 5000 digitized biopsies and achieved internal and external AUROC values of approximately 0.85 and 0.87, which translated into approximately 43% fewer reflex molecular tests while maintaining sensitivity. In a prospective deployment simulated study, it achieved an AUROC of 0.89 on new cases and substantially reduced biomarker reporting time [22]. This approach conserves tissue for comprehensive sequencing and shortens the turnaround time for initiating targeted therapy [22]. Similar AI approaches are being explored to predict other actionable alterations, such as anaplastic lymphoma kinase (ALK) or ROS proto oncogene 1 (ROS1) fusions, from morphology. However, their rarity requires larger training datasets [78] (Table 2).

**Table 2:** NSCLC digital pathology task overview

Subdomain	Input modality	External validation (centers/batches/scanners)	Clinical use/potential impact	Key references
Histologic subtyping	H&E whole-slide images (WSI)	Reported in some studies; details NR	Standardized subtyping; reduced subjective variability	[15, 20, 76, 79]
Molecular biomarker prediction	H&E WSI	Yes (multicenter, > 1 scanner; batch differences present)	~43% reduction in reflex molecular tests; tissue conservation; shorter TAT	[20, 22, 77]
Prediction of actionable fusions (ALK/ROS1) from morphology	H&E WSI	Larger-scale validation pending (NR)	Prescreening to optimize molecular testing	[78]
IHC quantification	IHC slides	Some cross-center/reader work (NR)	Standardized IHC scoring; supports immunotherapy decision-making	[21, 80]
Clinical integration and expert support	WSI/IHC plus reporting systems	Real-world deployment progressing (NR)	Provides explainable evidence to augment, not replace, experts	[28, 49, 51]

AI-based analysis of immunohistochemical slides yields more consistent protein-biomarker quantification than manual scoring [21, 80]. For example, in 2022 Wu and colleagues developed a deep learning system to score PD-L1 IHC in NSCLC. The model’s tumor-proportion scores showed strong agreement with pathologists, with a correlation coefficient of about 0.94, and improved inter-pathologist consistency [21]. Overall, AI in pathology enables precise subtyping of NSCLC by extracting detailed phenotypic information from routine slides [79]. These systems automate tumor classification, identify diagnostically relevant regions for review, and predict molecular markers without invasive procedures. Importantly, these tools are designed to support human experts rather than replace them [81]. With appropriate validation and regulatory approval, AI-augmented pathology can standardize diagnoses across centers and ensure accurate histologic and molecular characterization for each patient, which are foundations of personalized NSCLC care [15, 22].

Notwithstanding these advances, several limitations and sources of bias warrant emphasis.Across H&E based biomarker studies, external validation is inconsistent and subgroup calibration is seldom reported [82]. Many studies do not enforce patient level splits that separate sites and scanners at the partition stage, increasing the risk of leakage from acquisition related signatures [83]. Few reports include grayscale or stain normalized ablations, per site performance with confidence intervals, or negative region controls. Saliency maps are often presented without validity checks or quantitative evaluation. We recommend patient level splits that separate sites, explicit per site calibration, color and stain ablations, tumor only masking analyses, and stability tests across random initializations. These reporting elements help distinguish genuine morphologic associations from confounding factors [84].

In WSI pipelines, tile-level heatmaps often co-localize with staining or batch signatures, or tissue processing borders, instead of tumor morphology [85]. Without color or stain ablations, tumor-only masking, and cross-scanner stability tests, saliency can be misleading [86]. We recommend reporting the following: (i) color-space or stain normalization ablations with effect size and confidence intervals; (ii) negative-region controls, such as background and artifacts; (iii) concept-based validation, such as TCAV for gland formation, keratinization, or TIL density with bootstrap-tested significance; and (iv) slide-level counterfactuals, such as swapping stain or morphology exemplars, to test causal relevance of the explanation. Incorporate prototype-based or concept-bottleneck heads when feasible to improve faithfulness and auditability.

## Role of AI in prognosis prediction and risk assessment

### Integration and analysis of multiomics data

#### Rationale and data types

Prognosis improves when multiomics data that comprise genomics with mutations and copy number alterations, transcriptomics, methylomics, proteomics, and metabolomics are integrated with ctDNA, image-derived features from radiology and pathology such as radiomics and pathomics, and routine clinical variables [26, 87–89]. AI enables representation learning and discovery of cross modal interactions beyond linear additivity. These advances yield composite risk scores that better capture tumor biology and the host context [48].

#### Model classes and fusion strategies

Model classes and fusion strategies are summarized as follows. Approaches include penalized Cox models with learned embeddings [90]; deep survival models such as DeepSurv and DeepHit, Transformer based fusion for variable length longitudinal sequences [91]; and GNNs that capture pathway and interaction structure. Fusion can occur at the feature level, known as early fusion; at the decision level, known as late fusion; or at intermediate layers via cross attention [9, 91]. Pathway aware regularization improves interpretability by aligning learned representations with biological circuits [92]. To support model selection and reproduction, we summarize core architectural choices, hyperparameter sensitivity, and compute trade offs across these model classes [93]; see Table 3. Transformer based fusion requires careful specification of tokenization, sequence length and windowing, the number of layers and heads, the hidden width, and the attention variant. Training cost and inference latency scale with the token count and the network depth, which can constrain deployment in resource limited settings [94]. Practical mitigations include parameter efficient fine tuning with adapters or low rank adaptation, mixed precision training, quantization, pruning, and knowledge distillation [95]. For GNNs, the choice among convolutional families such as GCN, GraphSAGE, and GAT interacts with graph construction and neighborhood size, and excessive smoothing or excessive squashing can degrade performance [96]. For whole slide image pipelines, tile size and stride, stain normalization, and the attention pooling strategy strongly affect both accuracy and throughput. Across all classes, the most sensitive hyperparameters typically include the learning rate, batch size, weight decay, data augmentation, and early stopping criteria. We recommend reporting wall clock training time, GPU type and count, peak memory footprint, and per case inference time, and releasing exact configuration files and fixed random seeds to enable deterministic reruns [97].

**Table 3:** Multimodal AI for NSCLC: from diagnosis to personalized therapy

Model class	Inputs	Objective	Limitations	Data/compute demand	Key hyperparameters and implementation notes	Key refs
Penalized Cox with learned embeddings	Hand-crafted and learned representations	Time-to-event	Linear risk composition unless embeddings capture non-linearity	Low to moderate; CPU or single GPU	Regularization strength, embedding size, feature scaling; report convergence and proportional hazards checks	[90]
Deep survival models (DeepSurv, DeepHit)	Images/WSI/omics and clinical	Hazard/risk or discrete-time event probability	Calibration and transportability require care	Moderate; single to few GPUs	Learning rate and batch size, early stopping, label discretization for discrete-time, augmentation; report time-dependent AUROC and IBS	[91]
Transformer-based fusion	Longitudinal imaging, ctDNA, labs, notes	Sequence modeling of risk over time	Data-hungry; careful masking/temporal encoding needed	High; scales with sequence length and depth; memory bound	Token size and stride, sequence length and windowing, layers and heads, hidden size, attention variant; adapters or LoRA for efficient tuning; mixed precision and quantization for deployment	[91]
GNNs	Omics graphs; patient–feature graphs	Risk via structured message passing	Graph construction choices matter; scalability	Moderate; depends on graph density	Aggregation type, neighborhood size, number of layers; avoid oversmoothing; document graph build rules	[92]
Fusion strategies	—	—	Early needs harmonized preprocessing; Late may miss interactions; Intermediate more complex	Varies	Decision-level vs cross-attention; report latency and memory impact of fusion block	[9, 91]

#### Recent exemplars and effect sizes

Multimodal survival models that combine CT radiomics, WSI embeddings, mutational signatures, and clinical covariates typically increase the concordance index by 0.05 to 0.12 over clinical baselines and lower the integrated Brier score. External validations show good portability with calibration drift that is usually modest and amenable to recalibration [26, 51, 98, 99]. Early stage I to II studies that combine genomics and pathomics have identified high risk subgroups that gain an absolute 5% to 10% overall survival benefit from adjuvant therapy, whereas low risk groups may be candidates for de escalation of therapy [76, 100, 101].

#### Dynamic and longitudinal risk

Landmarking and joint models update risk after each assessment, including ctDNA kinetics, radiographic response, and laboratory trends. These approaches outperform static baselines on time dependent area under the curve and enable earlier escalation or de escalation of therapy [102–106]. Reinforcement learning policies for adaptive sequencing are promising but require prospective oversight and clearly defined safety constraints [107–109].

#### Scale, privacy, and fairness

Multi institutional training is essential to capture real world variability [110]. Federated learning (FL) enables training across sites without moving raw data, while secure aggregation, client side differential privacy, and auditable logs protect confidentiality [111–113]. Fairness audits should be routine, with subgroup calibration and utility metrics such as calibration within groups and equalized odds, and any remediation, including reweighting or adversarial debiasing, should be documented [79, 114].

#### Reporting checklist (prognosis)

Studies should: (1) pre register analysis plans; (2) use internal and external validation across sites and time;(3) report calibration plots and metrics, including area under the curve, concordance index, integrated Brier score, and DCA, with time dependent estimates where relevant; (4) account for competing risks; (5) report subgroup performance by sex, race or ethnicity, geography, and smoking status; and (6) release code, parameter files, and data dictionaries to enable reproducibility.

### Multimodal survival prediction models

AI driven prognostic models for NSCLC typically output a predicted survival time or a patient specific risk score. These outputs guide clinical decisions, such as whether to add adjuvant chemotherapy after surgery and how intensively to follow a patient [105]. Traditional models, such as tumor node metastasis staging, consider only a few variables, whereas AI models can integrate many features from imaging, pathology, and omics [26]. As a result, AI models demonstrate improved discrimination for survival stratification [100].

For example, image based AI has identified imaging features that correlate with outcomes independent of stage [115, 116]. In a pilot study, a deep learning model applied to pre treatment CT scans predicted overall survival in NSCLC, and the model derived risk scores separated patients into distinct prognostic groups. In that study, the AUROC was approximately 0.70, outperforming a model that used only clinical factors, which achieved approximately 0.60 [117]. Similarly, AI derived pathomic features from H&E slides have prognostic value. A 2023 study reported five year survival prediction with area under the curve values between 0.64 and 0.85, exceeding models based on tumor grade or stage alone [118]. Together, these approaches enable a digital prognostic assay built from routine diagnostic data [116]. Combining radiology based and pathology based AI predictors with clinical variables yields integrated survival models. Several institutions are evaluating AI based survival nomograms for NSCLC that output individualized survival probabilities. In 2023, Song and colleagues developed a nomogram that combined a deep learning radiomics signature derived from CT with clinicopathologic variables to predict progression free survival in stage IV EGFR mutant NSCLC treated with EGFR inhibitors. The model improved one year progression risk stratification [119].

Beyond static baselines, AI models can provide dynamic prognostic updates [103]. For example, serial imaging combined with ML can assess response trajectories and adjust survival predictions; this approach is known as dynamic risk prediction [40]. In practice, dynamic risk is modeled with sequence Transformers that consume tokens indexed by event or by visit, together with temporal and frequency attention [29]. Fourier layers capture long range periodicity, and wavelet blocks capture abrupt transients induced by treatment regimens [30–32]. Key hyperparameters to report include sequence length and windowing, the time embedding scheme, which may be absolute or relative, the number of layers and attention heads, and the masking strategy for irregular sampling.Multiple time point radiomic modeling in lung cancer shows that changes in tumor texture or other features after a few therapy cycles predict long term outcomes better than baseline features alone [120]. In addition, multi omics prognostic models have been applied in specific contexts, such as early stage NSCLC after surgery [89]. One AI model integrated gene expression profiles with clinical factors to predict which patients would benefit from adjuvant chemotherapy. It identified a subset of stage I patients at high risk of recurrence who experienced significantly improved survival with chemotherapy, illustrating the potential of AI for decision making based on prognostic stratification [89, 121].

**Fig. 2:**
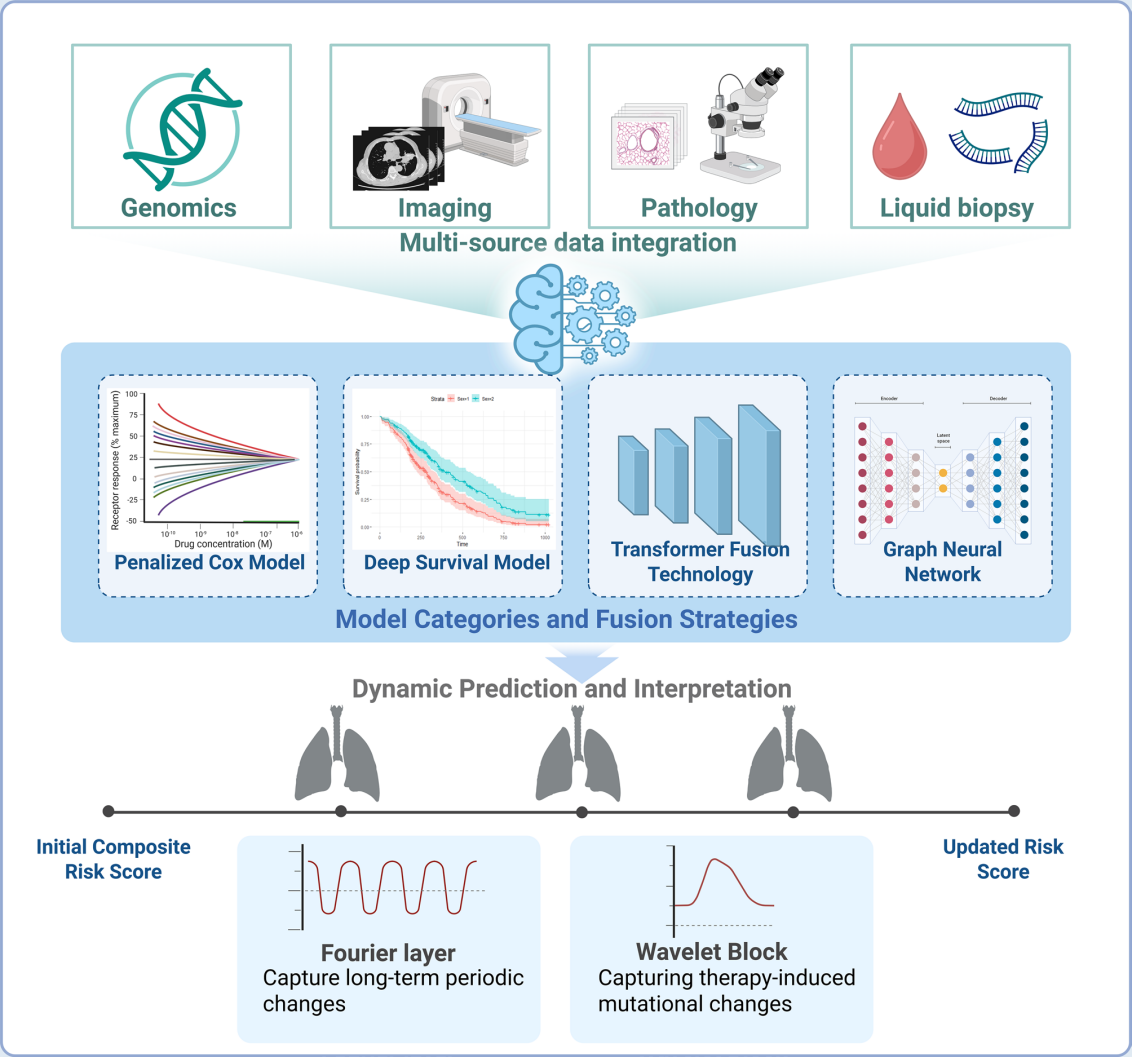
Multimodal data integration and fusion models for dynamic risk prediction in NSCLC

In summary, AI-based survival-prediction models—especially those built on multimodal data—are achieving higher accuracy and finer risk discrimination in NSCLC [26]. They hold promise for personalized risk assessments that inform patient counseling and rational treatment tailoring [38]. However, prospective validation remains essential [51]. Many published models still require rigorous external validation across diverse cohorts to ensure generalizability beyond their training sets [122]. As these models mature, they could be incorporated into practice via decision-support systems that, for example, flag a “high-risk” early-stage patient for closer follow-up or prompt consideration of novel adjuvant therapies [123] (Fig. 2). Despite these advances, important limitations and potential sources of bias remain. First, many studies rely on internal validation and lack temporally or geographically external test cohorts, and the reporting of calibration and DCA is inconsistent [124]. Second, patient level splits that separate sites and scanners are often missing, which can allow acquisition related signatures to inflate performance [83]. Third, dynamic models can inadvertently leak post baseline information into the target or comparator, introducing immortal time bias and overly optimistic estimates. Fourth, approaches to censoring, competing risks, and treatment switching vary widely, and concordance alone does not capture calibration over time. Finally, subgroup calibration by sex, age, ancestry, stage, and site is rarely presented, fairness analyses are uncommon, and most reports omit drift monitoring and change control plans [125].

## AI in treatment decisionmaking and personalised therapy

**Fig. 3:**
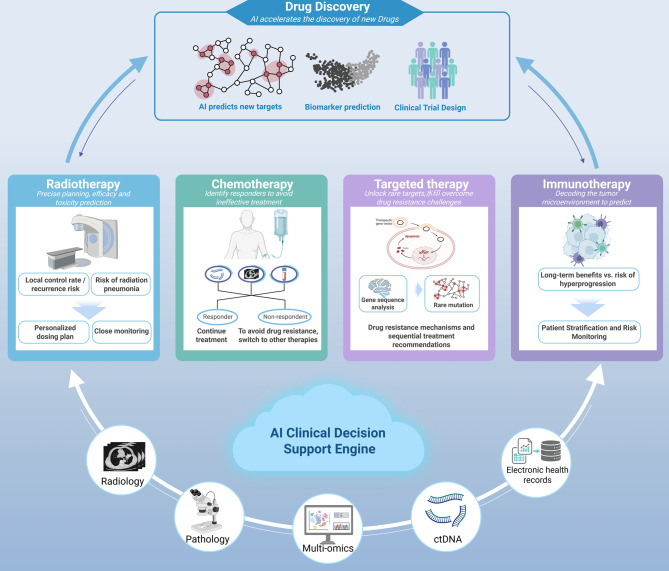
From multimodal evidence to action: AI guidance for radiotherapy, chemotherapy, targeted therapy, immunotherapy, and drug discovery in NSCLC

**Fig. 4:**
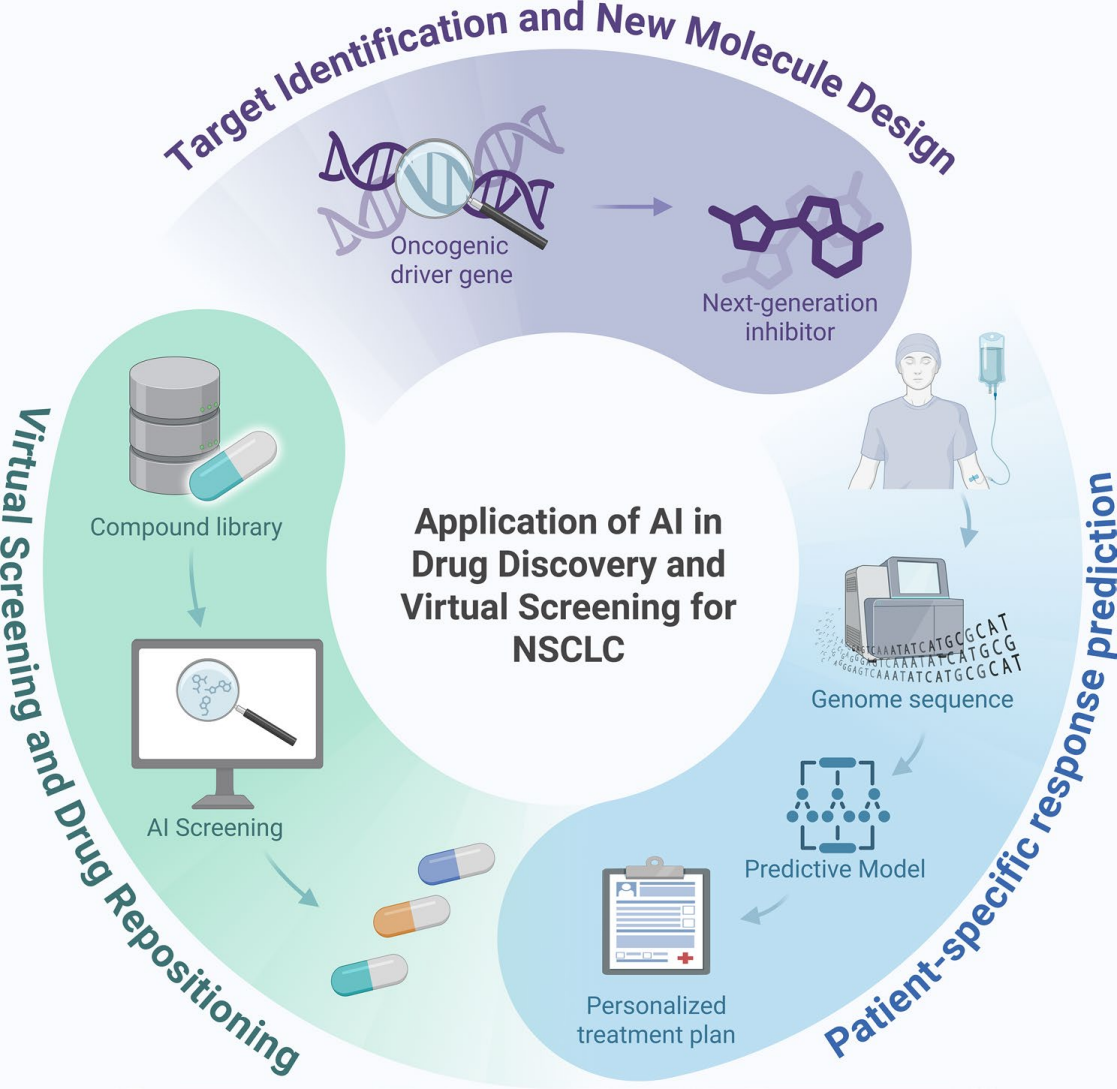
AI in NSCLC drug discovery: from target identification to patient-specific response prediction

AI for treatment decision making in NSCLC is moving beyond proof of concept and toward clinically consequential systems. These models integrate high dimensional, multimodal, and longitudinal data from radiology, pathology, multi omics and ctDNA, and electronic health records, producing calibrated, patient specific inferences that humans cannot reliably integrate at scale. Foundation and multimodal models learn unified representations that support response prediction and toxicity forecasting across therapies. Development and reporting should follow contemporary guidance on external validation, calibration, DCA, and deployment readiness. Critically, models should update risk over time by using serial imaging, circulating biomarkers such as ctDNA kinetics, and real world data streams. This dynamic updating better reflects disease trajectories and treatment effects in practice. When estimating individualized benefit, causal ML approaches can complement prediction to guide treatment escalation or deescalation. Reinforcement learning based adaptation is promising but requires explicit safety guardrails and prospective oversight. Against this backdrop, section “Multimodal survival prediction models” summarizes evidence for response and toxicity prediction across radiotherapy, chemotherapy, targeted therapy, and immunotherapy, and highlights actionable operating points and clinical utility;see Fig. 3. section “Response and toxicity prediction across modalities” reviews AI in drug discovery and virtual screening, where generative and structure aware approaches, for example AlphaFold supported pipelines, are accelerating target identification and lead optimization for NSCLC; see Fig. 4.

### Response and toxicity prediction across modalities

#### Radiotherapy

AI improves radiotherapy planning and predicts which patients are likely to benefit from radiation or experience treatment related harm [126]. For planning, recent methodological reviews advocate standardized and externally validated segmentation pipelines with transparent reporting of architectures, key hyperparameters and computational constraints, which enables reliable auto contouring and more consistent plans across sites, including resource limited centers [127–129]. For response prediction, radiomics based models identify imaging features associated with tumor radiosensitivity. For example, radiomic patterns on pre treatment CT have been linked to post radiotherapy local control and recurrence risk in NSCLC [130]. In one study, a radiomics signature predicted two year local recurrence after definitive chemoradiation with an AUROC of approximately 0.75, outperforming traditional stage based estimates [131]. AI models have also been developed to forecast radiation induced toxicities [38]. A recent multi institutional deep learning model combined dosiomics, radiomics, and clinical data to predict grade two or higher radiation pneumonitis in locally advanced NSCLC [132]; The integrated model achieved an external validation AUROC of approximately 0.80 and yielded well calibrated risk estimates. Similarly, ML models predict radiation related cardiac toxicity and pulmonary fibrosis by analyzing pre treatment scans together with dose volume parameters [133]. These tools could enable personalized radiotherapy by identifying patients with a high predicted pneumonitis risk who may benefit from alternative approaches or closer monitoring [134].

#### Chemotherapy

Predicting chemotherapy response in NSCLC remains challenging, yet AI is increasingly used to identify biomarkers of chemosensitivity [135]. ML models that use genomic signatures and circulating biomarkers have been explored to predict which tumors will respond to platinum based regimens [136]. Gene expression based chemotherapy response scores show potential for predicting response to neoadjuvant chemotherapy in resectable NSCLC and may help avoid futile treatment in non responders [121]. Radiomics has also been evaluated. A deep learning model using radiomic features distinguished responders from non responders to first line chemotherapy on baseline CT with modest accuracy of approximately 70% [137]. In addition, AI analysis of blood based biomarkers, for example serum N glycome changes, has been investigated to forecast efficacy [138]. Although no AI test for chemotherapy response is in routine clinical use, these early studies suggest tools that could guide selection between intensive chemotherapy and alternative treatments for individual patients [139].

#### Targeted therapy (EGFR/ALK inhibitors)

In oncogene driven NSCLC, the key question is not only whether a tumor harbors a targetable alteration but also how durable the treatment response will be [140]. AI models are being developed to predict outcomes with targeted therapies and to identify early in treatment which patients may need additional interventions. Among patients with EGFR mutant NSCLC who start EGFR tyrosine kinase inhibitors, only a subset achieve prolonged progression free survival, whereas others progress rapidly because of de novo resistance [141]. Two recent studies applied ML to baseline clinical and imaging data to predict short progression free survival with EGFR tyrosine kinase inhibitors, thereby flagging high risk patients who might benefit from upfront combination strategies [142]. In these studies, the models identified patients at high risk of early progression within six to nine months with reasonable accuracy. These predictions are clinically actionable. For a patient predicted to respond poorly to EGFR tyrosine kinase inhibitor monotherapy, an oncologist might add chemotherapy or a vascular endothelial growth factor inhibitor at treatment initiation, an approach that can improve outcomes but may increase toxicity [143]. Similarly, in ALK positive NSCLC, where multiple ALK inhibitors are available, AI models are being explored to predict which specific inhibitor a tumor is most likely to respond to based on biological differences derived from omics data [144, 145].

#### Immunotherapy

Because only about 20 to 30% of unselected NSCLC patients respond to immune checkpoint inhibitors, identifying reliable predictive biomarkers is essential l [146]. AI has been used to discover new biomarkers and to integrate multiple signals into more accurate composite predictors [38]. In computational pathology, AI models analyze the spatial organization of cells and immune infiltrates on H&E slides to infer tumor immune phenotypes [147]. Rakaee et al. developed Deep-IO, a deep learning model that predicts immune checkpoint inhibitor outcomes from pre treatment H&E slides. In a cohort of 958 patients with advanced NSCLC, the model score independently predicted response and survival and either outperformed or complemented PD-L1 and tumor mutational burden, with an area under the curve of 0.66 for objective response in external validation compared with 0.62 for PD-L1 at at least 50%. Combining the AI score with PD-L1 increased the area under the curve to 0.70 and identified responders with higher precision [148]. Radiomic profiling provides a complementary approach. CT features can distinguish hyperprogressive disease from durable benefit on programmed cell death 1 or PD-L1 inhibitors, and one classifier separated hyperprogressors from ordinary progressors with an area under the curve of about 0.87 [38]. Radiomic biomarkers that reflect heterogeneity or volume dynamics have also correlated with outcomes and with immune related adverse events. For example, a baseline CT signature predicted the risk of immunotherapy induced pneumonitis and enabled closer monitoring [149]. Multimodal models that integrate radiomics, PD-L1 expression, and ctDNA metrics have outperformed individual predictors for one year survival on immunotherapy [26]. By tracking temporal changes during treatment, deep learning models can detect response or progression earlier than the Response Evaluation Criteria in Solid Tumors and thereby prompt earlier treatment changes [102]. Overall, AI driven response prediction across modalities is moving NSCLC care toward personalized therapy by matching each patient to the strategy most likely to maximize benefit and minimize harm. Although many models remain experimental, some pathology based immunotherapy predictors are undergoing prospective evaluation with encouraging early results [148, 150].

### AI in drug discovery and virtual screening

Beyond clinical decision support, AI is reshaping the early phases of NSCLC drug development [38]. AI driven discovery applies computational algorithms to identify therapeutic targets, design novel molecules, and repurpose approved agents, often much faster than traditional laboratory screening [151]. NSCLC exhibits numerous genomic alterations and resistance mechanisms, creating many opportunities for new therapies; however, efficient discovery remains challenging. ML models, including deep generative approaches, learn from large chemical libraries and bioassay data to predict compounds that inhibit cancer targets or overcome resistance [152].

One important application is the discovery of next generation inhibitors against established oncogenic drivers [152]. Resistance to third generation EGFR tyrosine kinase inhibitors, such as osimertinib, often emerges through EGFR T790M and C797S mutations [153]. In 2024, Zhou and colleagues used a ML aided approach to identify CDDO-Me as a potential fourth generation EGFR inhibitor active against T790M mutant NSCLC. The model screened hundreds of candidates by learning structure–activity relationships, and it predicted CDDO-Me, which was later confirmed experimentally, to strongly suppress proliferation of T790M mutant NSCLC cells, including in xenograft models. This approach markedly narrowed the pool of candidates for laboratory testing, demonstrating how AI can accelerate lead compound discovery [152]. Similarly, in 2023, Zhang and colleagues used ML with support vector machines and random forests to design new small molecules targeting EGFR active site mutations, achieving external accuracy greater than 95% and guiding feature optimization, with R^2 approximately 0.93 between predicted and experimental activity. These AI designed compounds are now candidates for further preclinical development [154].

AI is used for virtual screening of existing libraries to identify repurposing opportunities in NSCLC [155]. ML algorithms predict drug target interactions and synergistic combinations by mining patterns in historical pharmacologic data [156]. For example, a platform analyzing transcriptomic profiles predicted that a Food and Drug Administration approved kinase inhibitor, not originally indicated for lung cancer, could have activity in KRAS mutant NSCLC; subsequent laboratory testing confirmed this prediction and led to a new clinical trial [157]. Additionally, deep learning models such as GNNs operating on molecular graphs have been used to predict compounds that may inhibit novel targets, for example KRAS G12C and MET exon 14 skipping, thereby guiding medicinal chemistry efforts [158]. In silico approaches also extend to immunotherapy, with neural networks proposing small molecules or peptides that modulate immune checkpoints or the tumor microenvironment [159].

Another emerging paradigm is the prediction of patient specific drug response enabled by AI [157]. Large cell line screens, for example profiling hundreds of compounds across dozens of lung cancer lines, generate data that AI uses to map genomic profiles to likely drug responses [160]. For example, the open source tool D3EGFR provides a deep learning based web server that predicts the sensitivity of EGFR mutant lung cancers to various tyrosine kinase inhibitors and their combinations [161]. As more cell line and organoid screening data accumulate, AI can match NSCLC tumors to optimal therapies, effectively conducting in silico clinical trials to prioritize treatments. This approach is particularly relevant for rare molecular subsets in which real world trials are difficult to conduct [157].

In summary, AI is accelerating NSCLC drug discovery on several fronts: identifying new candidates such as small molecule and biologic agents for resistance pathways; optimizing leads by predicting how structural changes affect activity and by using reinforcement learning to generate improved analogs; and repurposing or prioritizing agents for defined patient subgroups [162]. The success of AI discovered agents such as CDDO Me against EGFR T790M suggests a future in which AI augmented medicinal chemistry markedly shortens the timeline from target identification to effective therapy [154]. When coupled with rigorous wet laboratory validation, these approaches could expand the therapeutic arsenal for NSCLC, particularly for patients who have exhausted current options [162]. Close collaboration among computational scientists, chemists, and oncologists is essential to ensure that AI predictions are rigorously validated and translated into clinically viable drugs [139]. (Table 4) Despite this progress, important limitations and methodological conflicts remain. First, many studies use random or scaffold split validation, which allows near duplicate chemotypes to enter the test set and inflates performance; temporal splits and external assays from independent sources are needed [163]. Second, assay heterogeneity, batch effects, and differences in experimental conditions can confound activity labels, and few reports include orthogonal confirmation across multiple assay formats [164]. Third, generative models may propose molecules that are difficult to summarize, unstable, or outside the applicability domain; synthesizability metrics, retrosynthesis success rates, and medicinal chemistry review are rarely reported [165]. Fourth, docking and scoring functions used to label data or triage candidates are noisy and can bias learning, and enrichment should be benchmarked against strong baselines with decoys and property matched controls. Fifth, translation from cell lines to patients is limited by context mismatch, off target effects, and absorption, distribution, metabolism, and excretion and toxicity constraints; prospective, blinded screens and pre registered evaluations are uncommon. Sixth, uncertainty and calibration are seldom quantified, and studies frequently omit hit rate, precision at top k, and prospective enrichment metrics that would inform decision making [166].

**Table 4:** Prospective-facing summary of AI for NSCLC drug discovery and virtual screening

Study (first author, year)	Task/Goal	Key outcomes (quantitative)	Prospective/translational status	Key refs
Zhou, 2024	Identify 4th-gen EGFR inhibitor active against resistance	CDDO-Me suppressed T790M-mutant NSCLC growth (exact IC50/TGI NR)	Preclinical; candidate for further development	[152, 154]
Abramson (AlphaFold3), 2024	Complex structure prediction to inform docking/design	SOTA complex prediction (platform)	Integrated in pipelines; preclinical utility	[151]
Meller (PocketMiner), 2023	Predict cryptic binding pockets	Improved cryptic pocket detection	Design aid; upstream of med-chem	[158]
STTT review + platform exemplar	Repurpose FDA-approved kinase inhibitor for KRAS-mutant NSCLC	Activity confirmed preclinically	Prospective clinical evaluation underway	[155, 157]
Zhao, 2025	Evidential DL for drug-target interactions	Calibrated DTI improvement	Platform tool; supports prioritization	[156]

## Challenges, limitations, and ethics

### Data bias and quality

#### Heterogeneity and bias

AI performance in NSCLC depends strongly on data provenance [167]. Variation in CT acquisition parameters such as tube voltage kVp, current time product mAs, slice thickness, reconstruction kernel, and vendor, and differences in PET protocols such as uptake time and calibration, introduce batch effects that distort learned representations.Digital pathology preanalytic factors including fixation, processing, staining, and scanner optics and compression have similar effects [168]. Labels are often noisy because of interreader variability and evolving diagnostic criteria, and class imbalance caused by rare histologies and uncommon fusions predisposes models to majority class bias and to poorer performance in minority subgroups [169].

#### Governance and documentation

Robust governance should include dataset datasheets and provenance logs; capture of preanalytic metadata for pathology, including ischemic time, fixation, stain, scanner model, and International Color Consortium (ICC) profile, and for imaging, including Digital Imaging and Communications in Medicine (DICOM) tags, dose, and reconstruction kernel; and versioned curation pipelines with auditable trails [170]. Such documentation enables reproducibility and supports forensic analysis after deployment [171].

#### Standardization and harmonization

For radiomics, adhere to IBSI definitions and publish exact parameters and code; apply physics aware harmonization such as ComBat and kernel aware resampling, and verify stability on repeat scan datasets [172]. For pathology, implement stain normalization, scanner aware augmentation, and color deconvolution, and confirm cross scanner reproducibility through ring trials [173].

#### Label quality and learning strategies

Use rater agreement protocols and adjudication panels for key endpoints such as PD-L1 TPS near cut points [169]. Leverage weak supervision using MIL, self supervised pretraining, and active learning to maximize label yield. Address class imbalance with cost sensitive losses, calibrated resampling, and careful evaluation in minority strata while avoiding synthetic oversampling artifacts in texture rich tasks [174].

#### Generalizability, OOD behavior, and robustness

Report internal to external performance gaps explicitly. Decreases of five to ten points in the AUROC across scanners or across sites are common [49]. Characterize distribution shift using stress tests and challenge sets,covering covariate shift,changes in label prevalence, and concept drift. Implement out of distribution (OOD) detection with Uncertainty Quantification (UQ) to enable selective prediction [175]. Maintain model cards that document failure modes, subgroup caveats, and guardrails. In addition, conduct external validation with a locked pipeline. Use patient level splits that keep sites and scanners separate, and apply preprocessing learned only on development data. Report discrimination, calibration, and decision utility together, and provide subgroup results by site, scanner, sex, age, ancestry, stage, and smoking status; see section “Validation, regulation, and workflow integration” for execution details [28]. To improve transportability, embed physics inspired modules that encode signal priors, including FA for global frequency structure, WA for multiscale edges and transients, and low rank tensor decomposition to control capacity. Publish transform settings, low rank factors, and domain shift ablations [30–32].

#### Privacy and security

Anticipate risks of model extraction, membership inference, and inversion. Where feasible, use FL with secure aggregation and consider differential privacy for sensitive modalities such as whole slide imaging and electronic health records [176]. Perform threat modeling and penetration testing, and monitor for anomalous access and potential data exfiltration [177] (Table 5).

**Table 5:** Reporting and governance checklist

Item	Minimum requirement for publication	Recommended best practice (deployment-ready)	Metrics/evidence to report	Key references
Data provenance and metadata	Dataset datasheet; key imaging/pathology metadata (DICOM tags, stain/scanner)	Full provenance logs; versioned curation scripts; audit trails	Data dictionary; inclusion/exclusion flow; preprocessing parameters	[170, 171]
Standardization and harmonization	IBSI-conformant radiomics definitions; code/parameters shared	Physics-aware harmonization (ComBat, kernel-aware resampling); pathology stain normalization and scanner-aware augmentation; ring trials	Test–retest stability; cross-scanner/site reproducibility	[50, 172, 173]
Label quality	Labeler count and roles; consensus rules	Adjudication panels for key endpoints; near-threshold protocols (e.g., PD-L1)	Inter-rater agreement (κ/ICC); sensitivity analyses to relabeling	[169]
Class imbalance and fairness	Class distributions; basic subgroup metrics	Cost-sensitive learning; calibrated resampling; fairness audit (by sex/ancestry/site/scanner)	Calibration-within-groups; equalized-odds/TPR gaps with CIs	[49, 169, 174]
External validity and shift	At least one external test	Internal–external validation across sites/time; stress tests; challenge sets	AUROC/C-index, Brier/ECE; reported internal–external gap (expect 5–10 points); coverage vs. accuracy under selective prediction	[49, 175]
Uncertainty and OOD	—	UQ (ensembles/MC-dropout/evidential); OOD detection; selective deferral policy	Calibration plots; risk-coverage curves; deferral utility/DECISION curves (DCA)	[175]
Privacy and security	Ethics approval; de-identification	FL + secure aggregation; differential privacy (where feasible); pen-testing	DP ε/δ (if used); federation topology; security test report; access monitoring	[176, 177]

Systematic data pitfalls are pervasive in existing studies (Table 6). Common weaknesses include (i) single-center, small-sample cohorts that inflate internal discrimination but fail under domain shift [178]; (ii) subjective and inconsistent annotations, particularly at near-threshold endpoints such as PD-L1 at 1% and 50%, with minimal adjudication or measurement-error analysis; (iii) systemic case-mix and acquisition biases—including site/scanner fingerprints, stage distribution, smoking status, ancestry, and socioeconomic proxies—that models learn and amplify [179]; (iv) leakage risks (patient overlap across splits and refitting normalization on combined data) that overstate performance [180]; and (v) imbalanced outcomes (rare fusions and never-smoker subsets) that produce unstable thresholds and poor calibration in under-represented groups [181]. We recommend explicit internal–external reporting with locked pipelines; patient-level splits that keep sites and scanners separate; and joint reporting of discrimination, calibration, and decision-curve analysis, each stratified by site, scanner, sex, age, ancestry, stage, and smoking status [124].

**Table 6:** Data quality pitfalls, subgroup harms, and mitigation/reporting playbook

Pitfall	Typical manifestation in NSCLC AI	Potential clinical harm	Primary mitigation
Single-center, small-sample cohorts	High internal AUROC; drop on external sites/scanners	Mis-triage under domain shift; delayed or missed care	Multi-site curation; locked pipelines; internal–external validation
Inconsistent/subjective labels	Label noise; unstable thresholds; poor calibration	Overtreatment/undertreatment near cutoffs	Adjudication panels; near-threshold SOPs; κ/ICC tracking
Systemic case-mix bias	Subgroup TPR/FPR gaps; risk miscalibration	Disparate false negatives/positives; unequal benefit	Targeted sampling; reweighting; DRO; subgroup thresholds
Acquisition bias	Model keys on device/site signatures	Fragile transportability; scanner-specific failures	Physics-aware harmonization; stain normalization; ring trials
Class imbalance	Minority underperformance; unstable PPV/NPV	Missed rare actionable findings	Cost-sensitive loss; calibrated resampling; synthetic data with caution
Leakage	Inflated metrics; failure at deployment	Unsafe optimism	Patient-level splits; freeze preprocessing; audit trails
OOD/unseen shifts	Confidence mismatch; brittle predictions	Silent failures; unsafe automation	UQ + OOD detection; selective deferral; drift alarms
Privacy/security gaps	Data misuse; membership inference	Legal/ethical risk; loss of trust	FL+secure aggregation; DP where feasible; pen-testing

### Explainability and clinical trust

#### From saliency to semantics

Post-hoc saliency methods such as Gradient-weighted Class Activation Mapping, Integrated Gradients, and Layer-wise Relevance Propagation can highlight image regions that influence predictions; however, their faithfulness and stability remain limited without dedicated validation [182]. Where feasible, prioritize intrinsically interpretable designs, including concept-bottleneck models that detect clinical primitives such as spiculation, necrosis, and tumor-infiltrating lymphocyte density; generalized additive models with pairwise interactions (GA^2 M); prototype or nearest-neighbor reasoning; and case-retrieval systems that surface similar prior patients and outcomes [183].

#### Evaluating explanations

Quantify faithfulness, defined as sensitivity to counterfactual perturbations; quantify stability, defined as repeatability across runs; and quantify utility, assessed by whether clinicians make better decisions faster. Avoid explanation theater by pairing explanations with quantitative UQ and systematic error analyses [184]. In pathology, complement heatmaps with tile-level concept scores that align with pathologist vocabulary.

#### Uncertainty, calibration, and selective workflows

Distinguish aleatoric and epistemic uncertainty, and use deep ensembles, MC-dropout, evidential networks, or conformal prediction to provide well-calibrated confidence for each output [185]. Enable abstention in low-confidence or OOD cases, and route such cases to expert adjudication or confirmatory testing, for example reflex next-generation sequencing for equivocal EGFR prescreening and manual PD-L1 scoring near 1% or 50% [186].

#### Communication and documentation

Standardize report templates to list the model name and version, training domains, intended use, contraindications, uncertainty bins, and recommended actions. Maintain accessible factsheets and change logs for tumor boards and quality assurance committees [187].

### Validation, regulation, and workflow integration

#### Validation ladder

Progress deliberately from technical validation that uses cross validation and internal and external splits, to clinical validation through multicenter retrospective studies with prespecified analysis plans, and ultimately to demonstrations of clinical utility through prospective evaluations such as Developmental and Exploratory Clinical Investigations of DEcision support systems driven (DECIDE)-AI style pilots, stepped wedge or cluster trials, and, where feasible, Randomized Controlled Trials [188]. At each stage, report discrimination, calibration, DCA, and net reclassification compared with standard care [28].

High retrospective accuracy is necessary but not sufficient for clinical adoption [189]. Translation requires evidence across technical, clinical, and operational domains. First, demonstrate generalizability and calibration under domain shift on external datasets that differ by time, geography, vendor, and protocol [190]. Beyond discrimination, report calibration slope near 1.0, expected calibration error with a prespecified tolerance, and decision-curve analysis at prespecified thresholds with net benefit and linked actions [189]. Provide subgroup calibration by site, scanner, age, sex, race or ethnicity, stage, and smoking status, with confidence intervals [28]. Second, characterize uncertainty and OOD behavior and define selective output and deferral policies with predefined coverage and deferral triggers [191]. Route low-confidence or OOD cases to expert review or reflex molecular testing, and disclose the impact on net benefit and resource use [192]. Third, address workflow and interoperability through seamless integration with Radiology Information Systems and Picture Archiving and Communication Systems, DICOM Structured Reports and Segmentation, Fast Healthcare Interoperability Resources (FHIR), and Clinical Decision Support Hooks [193]. In shadow mode, quantify turnaround time, alert burden expressed as alerts per 100 cases, coverage–accuracy trade-offs, and re-review rates relative to standard procedures [194]. Fourth, set explicit service-level agreements and safety guardrails, including targets for end-to-end inference latency, failure rates, audit-log completeness, automatic escalation near decision thresholds such as PD-L1 1 and 50%, and human–AI collaboration with mandatory second confirmation for high-risk tasks such as emergency pulmonary embolism detection [195]. Fifth, manage lifecycle governance and change control under medical-device principles by defining drift triggers such as decreases in external AUROC beyond a preset margin or expected calibration error exceeding a threshold [196]. Establish recalibration and rollback plans with version auditing, monitor multicenter performance for adverse AI events, and track the balance between coverage, accuracy, and workload. Sixth, include fairness and economics in the evidence base by auditing in-group calibration and equalized-odds with confidence intervals, and by reporting time-to-treatment reductions, avoided unnecessary tests, budget impact, cost-effectiveness, and the sensitivity of net benefit to threshold choices [197]. Together, these standards enable a measurable transition from high-performing models to safe, effective, and efficient clinical tools [194].

#### Regulatory frameworks

For AI and ML based software as a medical device, align with the International Medical Device Regulators Forum risk frameworks; the United States Food and Drug Administration total product lifecycle principles and predetermined change control plans for learning systems; the European Union Medical Device Regulation and In Vitro Diagnostic Regulation and the EU AI Act; and the United Kingdom Medicines and Healthcare products Regulatory Agency change programme. Operate under a quality management system compliant with International Organization for Standardization 13485, risk management according to International Organization for Standardization 14971, software lifecycle standards International Electrotechnical Commission 62304 and International Electrotechnical Commission 82304–1, current cybersecurity guidance, and Good ML Practice [198]. Plan postmarket surveillance with real world performance monitoring and define explicit triggers for recalibration or rollback [199].

#### Clinical integration and interoperability

Embed AI into routine clinical systems and data pipelines, including DICOM Segmentation and Structured Report objects, Fast Healthcare Interoperability Resources Observation and DiagnosticReport resources, Clinical Decision Support (CDS) Hooks, and Health Level Seven International order and result messages. Define inference service level agreements, maintain auditable logs and user controls, and run shadow-mode pilots before go-live to quantify alert burden and false positive externalities [193].

#### MLOps in healthcare

Manage models as living systems. Maintain dataset and feature versioning; use gated continuous integration and continuous delivery deployments; run synthetic and real world regression tests; monitor for distribution shift; schedule recalibration and periodic reapproval; and maintain documented rollback plans. Define clear roles, responsibilities, and escalation paths for adverse AI events [58, 200].

### General limitations and comparability across studies

Most multimodal fusion studies summarized here are retrospective and frequently single center, and they report only marginal gains over unimodal baselines, which calls into question clinical significance without net benefit analysis and action linked thresholds [28]. For example, a systematic review by Yu and colleagues of externally validated imaging models found that most algorithms performed worse on external datasets, highlighting the gap between internal discrimination and transportability [201]. In digital pathology, a 2024 meta analysis reported variable accuracy and frequent risk of bias, and a public audit of commercial products showed that only about forty percent had peer reviewed external validation, underscoring limited generalizability [82]. Even in promising cases such as the EAGLE pathology system for EGFR prescreening, internal and external AUROC values were about 0.85 and 0.87, and a prospective silent evaluation reached 0.89; however, these gains require translation into net benefit, explicit decision thresholds, and measurable reductions in time to treatment to establish clinical value [202]. Across prognostic models, reporting of calibration and DCA remains inconsistent, despite long standing guidance that clinical usefulness should be expressed as net benefit across clinically relevant thresholds [203]. Methodological pitfalls also persist, including potential leakage of post baseline information in dynamic models that leads to immortal time bias, heterogeneous handling of censoring, competing risks, and treatment switching, and insufficient subgroup calibration by sex, age, ancestry, stage, and site. Finally, cross paper comparisons are often not directly comparable because datasets, endpoints, preprocessing, and evaluation metrics differ, which limits interpretation of reported incremental gains unless studies share harmonized benchmarks, analysis plans, and reporting checklists such as TRIPOD+AI and DECIDE AI. Together, these observations support prospective multicenter evaluations with preregistered analysis plans, external and temporal test cohorts, decision relevant metrics such as net benefit and reclassification, and routine subgroup and site specific calibration to demonstrate durable generalizability and practical clinical impact.

### External validation and calibration

Execution. Predefine the full pipeline and keep it fixed during testing [204]. Use external cohorts that differ in geography and time and that span multiple vendors and protocols. Fit preprocessing only on development data and apply it unchanged to external data. Enforce patient level splits that separate sites and scanners. Report AUROC and area under the precision recall curve with 95% confidence intervals [205]. Report calibration in the large, the calibration slope and intercept, smooth calibration curves, and expected calibration error. Provide DCA across prespecified thresholds and report the net reduction in interventions at the chosen operating point. For survival outcomes, report the concordance index (C index), time dependent AUROC, and the integrated Brier score, and account for competing risks. Aim for at least 100 events and 100 non events for binary outcomes and at least 200 events for survival, or use internal external cross validation when event counts are limited. Follow TRIPOD plus AI and DECIDE AI for study planning and reporting [28].

Common pitfalls include tuning on the external set, refitting normalization on combined data, mixing patients across sites, altering class priors without recalibration, reporting discrimination without calibration or decision analysis, and omitting subgroup calibration by site, scanner, sex, age, ancestry, and stage. Preregistration, harmonized reporting templates, and routine stratification by site and subgroup help mitigate these risks [28].

## Future outlook: integration, interpretability, and equity

**Fig. 5:**
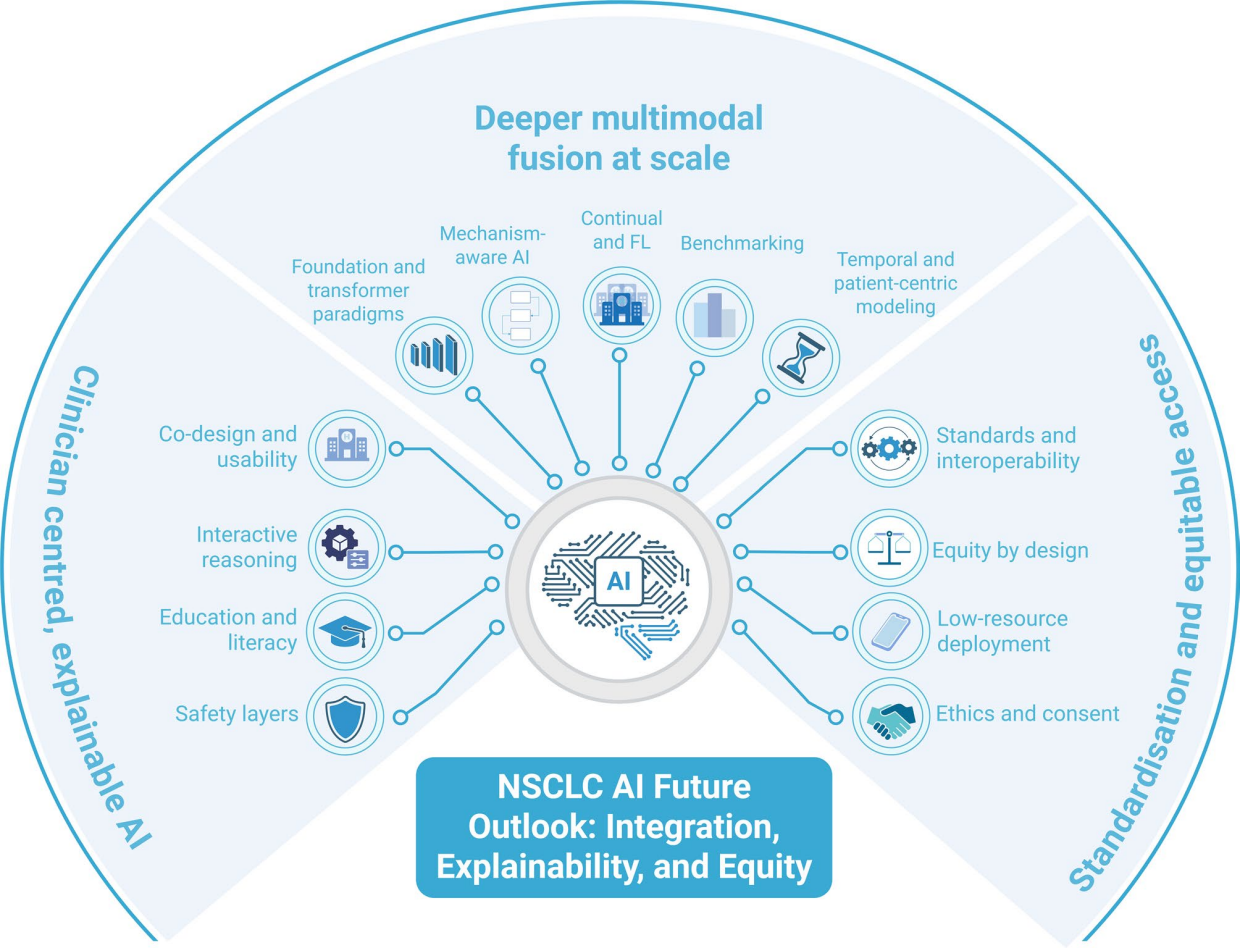
NSCLC AI future outlook: integration, explainability, and equity

Realizing the clinical promise of AI in NSCLC requires a shift from isolated single task models to an integrated clinician centered ecosystem that is scalable, interpretable, and equitable (Fig. 5). Foundation and cross modal Transformer backbones trained on diverse medical corpora can unify signals from imaging, pathology, multi omics and ctDNA together with electronic health records. The resulting shared representation supports NSCLC specific fine tuning for diagnosis, biomarker inference, risk trajectories, and treatment ranking. Beyond static baselines, temporal and patient centric modeling that links longitudinal imaging and ctDNA kinetics with digital twin simulations can update risk in real time and anticipate counterfactual treatment responses. Mechanism aware and causal methods align predictions with biology and estimate individualized benefit under confounding. Continual and FL keep models current as scanners, protocols, and therapies evolve while preserving privacy. Successful translation depends on clinician co design, usable explanations, and layered safety that includes uncertainty awareness and OOD aware abstention. It also depends on training in AI literacy and on routine evaluation of cognitive load and time to decision. Standards and equity are essential. Priorities include interoperability through the IBSI, DICOM Structured Report and Segmentation, FHIR, and Clinical Decision Support Hooks; benchmarking on open multicenter data sets; proactive fairness auditing with remediation; and pathways for deployment in low resource settings with clear consent and governance under emerging regulations.

### Deeper multimodal fusion at scale

#### Foundation and transformer paradigms

The field is converging on foundation models trained on diverse medical corpora, including CT and positron emission tomography, WSI, clinical notes, and structured laboratory data, paired with cross modal Transformers that learn joint latent spaces [206]. Fine tuning for NSCLC can produce unified outputs such as diagnosis, biomarker inference, risk trajectories, and ranked treatment options from a shared backbone [15]. Self supervised and weakly supervised objectives, including masked modeling and contrastive pairing of image, omics, and text, reduce labeling burden and improve transfer across institutions [20]. Concretely, foundation models tuned for NSCLC typically comprise three components. The first is a modality specific tokenizer, for example three dimensional patch embedding for CT, a tile encoder for WSI, a gene set projector for omics, and a text encoder for clinical notes. The second is a shared Transformer with between twelve and forty eight layers and between twelve and twenty four attention heads, connected by cross attention bridges for intermediate fusion [29]. The third is a personalization layer using adapters and low rank adaptation (LoRA) for site specific adaptation. For longitudinal use, FA or WA blocks can be inserted into temporal layers to couple slow trends and abrupt shifts [30–32]. We recommend that future studies report the number of layers, the number of attention heads, the hidden size, the token size and stride, the attention variant, the parameter count, the pretraining corpus and modalities, and the adapter rank.

#### Temporal and patient-centric modeling

Move from snapshots to trajectories. Use sequence models to represent longitudinal imaging, ctDNA kinetics, laboratory trends, and therapy timelines, and update risk in real time [103]. Combine these models with digital twin constructs that simulate counterfactual responses under alternative regimens and schedules, enabling “what if” exploration during tumor boards [207].

#### Mechanism-aware AI

Integrate pathway knowledge and causal constraints to reduce spurious associations by using pathway regularized networks, graph causal models that link radiomic heterogeneity to hypoxia and immune evasion programs, and joint models of tumor and host interactions [208]. For drug response modeling, estimate individualized treatment benefit with treatment effect methods such as uplift modeling, causal forests, and targeted maximum likelihood estimation, and explicitly account for confounding through appropriate adjustment or identification strategies [209]. This section’s discussion of causal forests, uplift modeling, and mechanism-aware models is reiterated in the Conclusion as a key set of emerging directions for individualized therapy response prediction.

#### Continual and FL

Anticipate continual updates as scanners, protocols, and therapies evolve [210]. Combine federated training with privacy preserving analytics to keep models current across networks while respecting local data governance requirements [112]. Distribute personalization layers using lightweight adapters that align models to site specific distributions without modifying the shared backbone [206].

#### Benchmarking

Establish open, multicenter NSCLC benchmarks that span multiple modalities and endpoints, including pathologic complete response, major pathologic response, event free survival, progression free survival, overall survival, and toxicity. Use standardized preprocessing and transparent protocol documentation, and maintain clearly separated training, validation, and test cohorts with temporally separated external tests to enable fair head to head comparisons and reproducibility [210].

### Cliniciancentred, explainable AI

#### Co-design and usability

Co-develop solutions with radiologists, pathologists, oncologists, and nurses so that outputs align with clinical mental models, including risk categories with associated confidence, flags tied to clinically relevant thresholds such as PD-L1 near one percent or fifty percent, and concise rationales linked to recognizable clinical features [65]. Evaluate cognitive load and time to decision in simulated tumor boards and iteratively refine the user experience [34].

#### Interactive reasoning

Provide what if and counterfactual tools, link to exemplar cases, and allow threshold adjustment based on patient preferences. Enable selective automation by fully automating low risk, high confidence tasks while requiring human approval for high risk or low confidence outputs [211].

#### Education and literacy

Incorporate AI literacy into oncology training, covering uncertainty interpretation, calibration, fairness trade offs, and the limits of generalization. Define competencies and credentialing, including safe overrides and adverse event reporting [212].

#### Safety layers

Embed multilayer safeguards, including abstention based on OOD detection and UQ, fail safe defaults, automatic escalation for biomarker predictions near decision thresholds, and drift alarms that notify stakeholders before clinically meaningful degradation [213].

### Standardisation and equitable access

#### Standards and interoperability

Consolidate around IBSI for radiomics, adopt common staining and scanning protocols for digital pathology, and ensure interoperability through DICOM SR and DICOM SEG, FHIR, and CDS Hooks. Publish reference implementations and open test suites to promote reproducibility [15, 50].

#### Equity by design

Proactively include underrepresented populations and sites in training and in external validation. Report calibration within groups and equalized odds by sex, ancestry, socioeconomic status, and geography [49]; When disparities are detected, apply remediation through reweighting, domain specific adapters, or targeted data acquisition.

#### Low-resource deployment

Optimize for edge inference, minimize dependencies, and support offline operation. Provide tiered models matched to local infrastructure, and consider pooled procurement and public private partnerships to reduce costs [214].

#### Ethics and consent

Clarify consents for secondary use, empower governance bodies to evaluate cross-border data flows and legal alignment, and reinvest benefits from AI deployments to improve access and outcomes in the communities that contributed data [215].

Verified explainability should be treated as a first-class requirement. Foundation and cross-modal models for NSCLC should be deployed with verified explanations as first-class outputs, audited according to the minimum evidence package, and accompanied by site-level adapters that maintain both prediction and explanation stability. Prospective evaluations should include human-factors endpoints and automation-bias monitoring. Regulatory dossiers should treat explanation verification with the same importance as discrimination and calibration.

## Conclusions

AI is moving from proof of concept tools to clinically consequential systems across the NSCLC continuum [15]. Beyond matching specialist level accuracy on narrow tasks, the distinctive value of AI is to integrate high dimensional, multimodal, longitudinal information from radiology, pathology, multi omics, and electronic health records into calibrated, individualized inferences that humans cannot reliably integrate at scale [206]. When properly validated and deployed, AI can improve timing, including earlier detection and risk stratification; targeting, through more precise therapy selection with less futile toxicity; and throughput, by enabling standardized and efficient workflows that return clinician time to complex decisions [210]. Current evidence supports AI as an adjunct that enhances screening fidelity, histologic and molecular subtyping, prognostication, and prediction of treatment response and toxicity. However, heterogeneity in external performance, domain shift across scanners and laboratories, and variability in preanalytics mean that models should not be judged by AUROC alone; calibration, transportability, uncertainty awareness, and clinical net benefit are also required [15]. In practice, the most useful systems generalize across settings with bounded performance loss, disclose uncertainty through selective prediction and abstention, and explain recommendations in clinically meaningful terms such as spiculation, necrosis, tumor infiltrating lymphocytes, and dose and volume drivers to support expert oversight [211, 216].

Causal and mechanism-aware modeling is a key emerging area central to individualized therapy-response prediction [217]. Causal forests and uplift modeling estimate individual treatment effects under confounding and heterogeneity [218]. Mechanism-aware representation learning encodes biological and physical priors, improving transportability, interpretability, and safety under distribution shift [219]. Clinical credibility requires multicenter evaluation with preregistered analysis plans, geographically and temporally external test cohorts, decision-relevant metrics such as net benefit and reclassification, and calibration reported within subgroups and by site [28]. Prospective pilots and pragmatic trials that embed these methods in tumor-board workflows can translate predictions into actionable strategies that balance benefit and risk for each patient.

Looking forward, three inflection points stand out: first, multimodal foundation models tuned for NSCLC should replace fragmented single task pipelines and produce unified outputs from a shared backbone with parameter efficient site personalization; second, causal and longitudinal modeling should become standard by combining treatment effect estimators with dynamic risk models that update after each imaging assessment, ctDNA measurement, or laboratory test; third, AI derived computable biomarkers should advance along formal qualification pathways, progressing from analytical validity and multicenter clinical validity to prospective demonstrations of clinical utility that inform trials, guidelines, and reimbursement [103, 118, 206, 209, 220]. Clinical translation requires an explicit pathway. We recommend a validation ladder that begins with internal and external splits, advances to preregistered multicenter retrospective studies, and culminates in DECIDE AI prospective evaluations and pragmatic trials where feasible [28, 214, 221]. At every stage, report calibration and DCA alongside discrimination, define operating points tied to actions, and prespecify recalibration plans [60, 222]. Deployment should follow Software as a Medical Device principles with postmarket surveillance, drift monitoring, rollback triggers, and change control plans for learning systems [223]. In summary, beyond accuracy and calibration, explanations verified for faithfulness, stability, and clinical utility, integrated with uncertainty-aware selective workflows, are essential for adoption in high-stakes NSCLC decisions. Our proposed minimum evidence package facilitates the implementation of this requirement.

## Data Availability

All data for this study have been provided.

## Abbreviations

NSCLC: Non-small cell lung cancer
LDCT: Low-dose computed tomography
EGFR: Epidermal growth factor receptor
AI: Artificial intelligence
ML: Machine learning
CT: Computed tomography
MIL: Multiple instance learning
PD-L1: Programmed Death Ligand 1
H&E: Hematoxylin and Eosin
FA: Fourier attention
WA: Wavelet Attention
CtDNA: circulating tumor DNA
GNNs: Graph Neural Networks
AUROC: rea Under The Receiver Operating Characteristic Curve
PET-CT: Positron Emission Tomography-Computed Tomography
MRI: Magnetic Resonance Imaging
IBSI: Image Biomarker Standardisation Initiative
LoG: Laplacian of Gaussian
DCA: Decision Curve Analysis
IHC: Immunohistochemistry
WSI: Whole Slide Images
TPS: Tumor Proportion Score
ALK: Anaplastic Lymphoma kinase
ROS1: ROS proto oncogene 1
FL: Federated Learning
UQ: Uncertainty Quantification
OOD: Out-Of-Distribution
ICC: International Color Consortium
DICOM: Digital Imaging and Communications in Medicine
FHIR: Fast Healthcare Interoperability Resources
